# Supplementary material for: Tumor-Derived Exosomal miR-143-3p Induces Macrophage M2 Polarization to Cause Radiation Resistance in Locally Advanced Esophageal Squamous Cell Carcinoma
Source: Int J Mol Sci. 2024 May 31;25(11):6082. doi: 10.3390/ijms25116082 (PMC11172887; doi:10.3390/ijms25116082)
Supplement: Supplementary file 1 [file ijms-25-06082-s001.zip › ijms-2994960-SI.pdf]

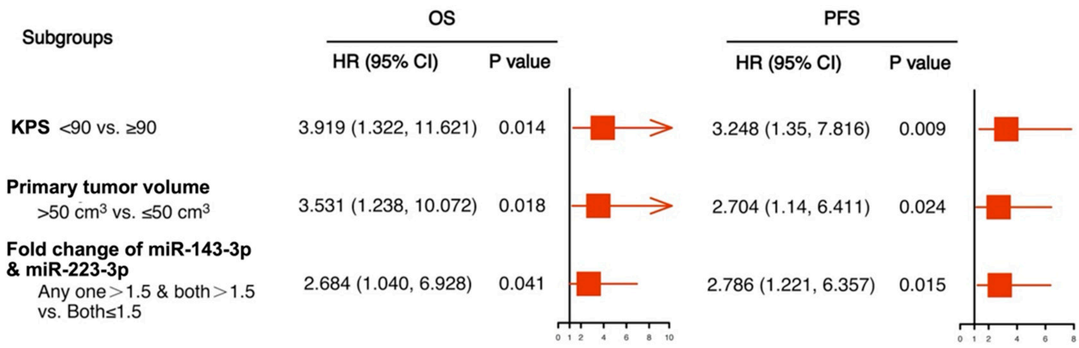


**Figure S1.** Results of multivariate Cox analysis for overall survival (OS) and progression-free survival (PFS). KPS, Karnofsky Performance Status; HR, hazard ratio; CI, confidence interval.

**Table S1. Cohort characteristics of six locally advanced esophageal squamous cell carcinoma patients**

| | Progression-free group (N = 3) | Progression group (N = 3) |
|---|---|---|
| **Age (year), Medium** | 64.3 ± 6.1 | 60.0 ± 6.2 |
| **KPS** | | |
| ≥90 | 1 | 1 |
| <90 | 2 | 2 |
| **Stage (AJCC 6$^{th}$)** | | |
| I-II | 1 | 0 |
| III-IV | 2 | 3 |
| **Location** | | |
| Cervical and upper | 1 | 1 |
| Middle and lower | 2 | 2 |
| **Length** | | |
| ≤5 cm | 1 | 1 |
| >5 cm | 2 | 2 |

**Table S2. Primers used in this study**

| Primer Name | Primer Sequence | |
|---|---|---|
| miR-143-3p | UGAGAUGAAGCACUGUAGCUC | |
| miR-181a-5p | ACCACUGACCGUUGACUGUACC | |
| miR-223-3p | UGAGAUGAAGCACUGUAGCUC | |
| miR-337-3p | UGUCAGUUUGUCAAAUACCCCA | |
| iNOS | Forward primer | GTTCCAGATGAATACTGGCAGTC |
| | Reverse primer | GCAACTGAACACTATCTTTCCCT |
| CD80 | Forward primer | CTCCCATCCTGGGCCATTAC |
| | Reverse primer | CAGGGCGTACACTTTCCCTT |
| IL1B | Forward primer | ATGATGGCTTATTACAGTGGCAA |
| | Reverse primer | GTCGGAGATTCGTAGCTGGA |
| Arg1 | Forward primer | GTGGAAACTTGCATGGACAAC |
| | Reverse primer | AATCCTGGCACATCGGGAATC |
| CD206 | Forward primer | GGGTTGCTATCACTCTCTATGC |
| | Reverse primer | TTTCTTGTCTGTTGCCGTAGTT |
| IL10 | Forward primer | CCTCCGTCTGTGTGGTTTGAA |
| | Reverse primer | CACTGCGGTAAGGTCATAGGA |
| β-actin | Forward primer | GGCGGCACCACCATGTACCCT |
| | Reverse primer | AGGGGCCGGACTCGTCATACT |
